# Remote Evaluation of Parkinson's Disease Using a Conventional Webcam and Artificial Intelligence

**DOI:** 10.3389/fneur.2021.742654

**Published:** 2021-12-23

**Authors:** Mariana H. G. Monje, Sergio Domínguez, Javier Vera-Olmos, Angelo Antonini, Tiago A. Mestre, Norberto Malpica, Álvaro Sánchez-Ferro

**Affiliations:** ^1^HM CINAC (Centro Integral de Neurociencias Abarca Campal), Hospital Universitario HM Puerta del Sur, HM Hospitales, Madrid, Spain; ^2^Department of Neurology, Northwestern University Feinberg School of Medicine, Chicago, IL, United States; ^3^LAIMBIO, Laboratorio de Análisis de Imagen Médica y Biometría, Universidad Rey Juan Carlos, Madrid, Spain; ^4^Parkinson and Movement Disorders Unit, Department of Neurosciences (DNS), Padova University, Padova, Italy; ^5^Division of Neurology, Department of Medicine, Parkinson's Disease and Movement Disorders Centre, The Ottawa Hospital Research Institute, The University of Ottawa Brain Research Institute, Ottawa, ON, Canada; ^6^Movement Disorders Unit, Neurology Department, Hospital Universitario 12 de Octubre, Madrid, Spain

**Keywords:** Parkinson's disease, kinematics, webcam, telemedicine, artificial intelligence and bio-inspired algorithms

## Abstract

**Objective:** This study aimed to prove the concept of a new optical video-based system to measure Parkinson's disease (PD) remotely using an accessible standard webcam.

**Methods:** We consecutively enrolled a cohort of 42 patients with PD and healthy subjects (HSs). The participants were recorded performing MDS-UPDRS III bradykinesia upper limb tasks with a computer webcam. The video frames were processed using the artificial intelligence algorithms tracking the movements of the hands. The video extracted features were correlated with clinical rating using the Movement Disorder Society revision of the Unified Parkinson's Disease Rating Scale and inertial measurement units (IMUs). The developed classifiers were validated on an independent dataset.

**Results:** We found significant differences in the motor performance of the patients with PD and HSs in all the bradykinesia upper limb motor tasks. The best performing classifiers were unilateral finger tapping and hand movement speed. The model correlated both with the IMUs for quantitative assessment of motor function and the clinical scales, hence demonstrating concurrent validity with the existing methods.

**Conclusions:** We present here the proof-of-concept of a novel webcam-based technology to remotely detect the parkinsonian features using artificial intelligence. This method has preliminarily achieved a very high diagnostic accuracy and could be easily expanded to other disease manifestations to support PD management.

## Introduction

Bradykinesia, defined as the slowness of movement and decrement in amplitude or speed (or progressive hesitations/halts) in continuous movement, is the most relevant clinical motor feature of Parkinson's disease (PD) (1). For its evaluation, the clinicians analyze the multiple aspects of movement, such as amplitude, speed, fatigue, and arrests when executing a motor task. Typically, a clinician integrates all these features. The best example is its rating into a single severity score of different bradykinesia tasks part of the Movement Disorders Society-sponsored revision of the Unified Parkinson's Disease Rating Scale motor subscale (MDS-UPDRS part III). This scale is the most used standard evaluation of motor function in PD (2) with a high test–retest reliability and inter-and intra-rater reliability (3, 4). However, it is an ordinal scale with only five discrete categories, and often its accuracy can be compromised due to the subjectivity of the assessment and the difficulty to detect the subtle changes in the consecutive time points.

To accurately quantify and analyze the motor performance of the patients with PD, the technology-based tools, such as the wearable sensors composed of accelerometers and gyroscopes can be used (5). These objective measurement tools can overcome the subjective and non-linear measures resulting from the clinical ratings (6). Additionally, they can be used to analyze the motor status of the patient in the home setting (7).

The optical motion capture systems based on video processing can also be used to study motor performance (8, 9). Specifically, some video-based systems are developed for the automated assessment of the upper limb movement in the patients with PD (10). These systems included cameras combined with the colored and reflective markers, bare hand tracking by the depth-sensing devices that traced the upper limb movement while performing the MDS-UPDRS part III bradykinesia tasks (11–13). These systems are traditionally used in a lab setting and have not yet been transitioned to the home environment. With the surge of telemedicine and remote consultation, there is a need for the supportive tools that permit an objective evaluation of movement remotely.

In this work, we propose a markerless video-based motion method to prove the concept that video-based objective classification of PD motor function based on bradykinesia is possible using a standard laptop webcam and an artificial intelligence algorithm. This analysis provides an ideal proof-of-concept for capturing bradykinesia of a patient with PD remotely while using an accessible, standard webcam video-camera.

## Methods

### Subjects

We recruited a consecutive cohort of 22 patients with PD and 20 healthy subjects (HSs). The eligible patients (i) had a PD diagnosis in the preceding 5 years according to the UK Brain Bank Clinical Criteria (14), further supported with (ii) a PET-^18^FDopa neuroimaging. We excluded the HSs in the presence of personal history, and first- and second-degree family history of any movement disorder (i.e., tremor or parkinsonism), and any known condition that could affect motor performance of the upper limbs. The demographic characteristics were assessed for both the groups, such as handedness (Laterality Preference Index, LPI) (15). An independent dataset containing *N* = 12 videos (six PD and six HSs) were also included as validation cohort for the test. The Ethics Committee of HM Hospitales approved the study protocol (protocol number: 18.05.1245-GHM). The participants provided the written informed consent before participating in the study.

### Clinical and Quantitative Motor Assessment

The participants were always evaluated after overnight *off* medication, and clinical evaluation included a motor assessment performed by two trained specialists (MHGM and ASF) using the MDS-UPDRS Part III. To evaluate the concurrent validity of the new method with other objective tests, motor performance was also evaluated with objective measures using the inertial measurement units (IMUs) (Kinesia^TM^ One system; Great Lakes Neurotechnologies Inc., Cleveland, OH, USA) (16). We quantified the motor function while performing the MDS-UPDRS-III bradykinesia upper limb tasks (finger tapping, hand movements, and pronation and supination movements of the hand). For that, the IMU was placed on the index finger. Output data from Kinesia^TM^ is a continuous score from 0 (less) to 4 (maximum) impairment.

### Video Data Collection

The participants were recorded with a computer webcam (640 × 426 pixels at 30 fps). During the examination, the participants rested their elbow on an armchair, and the camera was adjusted such that the hand and forearm were always present in the field of view. The participants were instructed to perform the MDS-UPDRS III bradykinesia upper limb tasks (finger tapping, hand movements, and pronation and supination movements of the hand) in front of the camera. Each task was performed three times with each hand separately (i.e., single-hand tasks, named unilateral motor tasks), and with both hands simultaneously (i.e., two-hand tasks, named bilateral motor tasks). For the normalization purposes, for each task the subjects were asked to stay in a certain position for a few frames. In the finger tapping and hand movements tasks, the patients were asked to do a maximum aperture and closing of the fingers or hand. In the pronation and supination movement of the hand task, the subjects were asked to extend the arm with the palm down and do the maximum supination movement. Each video sample was restricted to 12 s.

### Image Analysis

**Figure 1:**
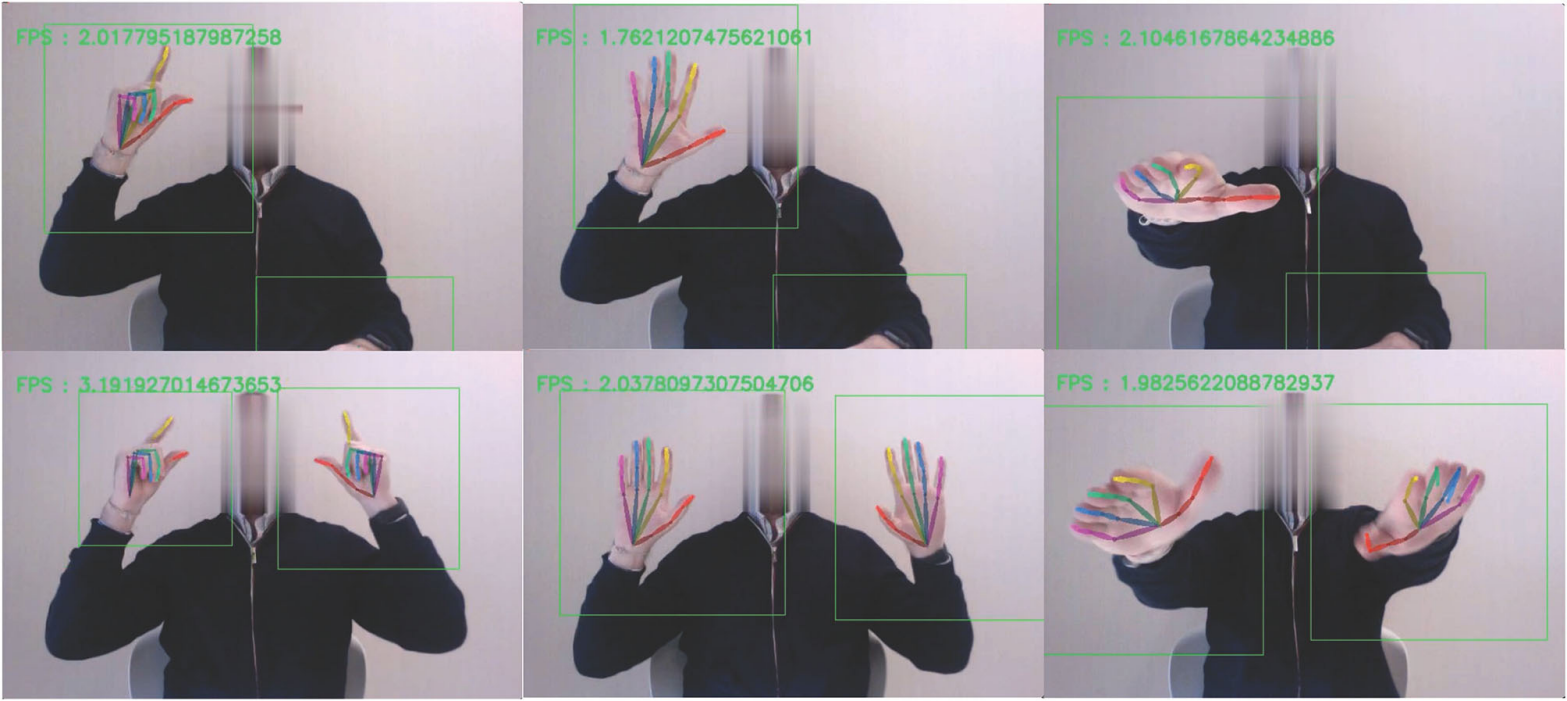
Video-capture motion. Example of finger tapping, hand movement, and pronation supination movement of the hand while performing the single-hand **(upper)** and two-hand **(bottom)** motor tasks. The bounding box is represented in green. The color markers over the hands represent specific landmarks extracted by OpenPose.

The video frames were processed with a Single Shot MultiBox Detector (SSD) network trained to detect the hands in real-time using the EgoHands dataset (17). The output of the SSD is a series of bounding boxes each marked with the probability of it containing a hand. The algorithm detects each hand and processes each of the two bounding boxes separately. To refine the detection process, we introduced some post-processing rules depending on the task. For single hand tasks, we selected the highest-ranking bounding box on the side required by the task, and for the two-hand tasks, we selected the highest-ranking bounding box on each side. We also performed a temporal correction. If the probability of a certain bounding box was not higher than its probability in the previous frame, we keep the previous bounding box. This pre-process ensures a correct and efficient detection of hands due to a varied background of the videos. After the bounding boxes for the hands were computed for all the frames, these boxes were cropped and processed by a second CNN model named OpenPose (18), to detect the joints of the hands (Figure 1). The specific landmarks extracted by OpenPose for each hand are shown in Supplementary Figure 1. From these landmarks, we select specific key-points to generate time curves to describe each task. To compensate for the camera distance and the size of the hand in the amplitude measurements, we normalized the measurements to the maximum amplitude. For finger tapping, the Euclidean distance (in pixels) between the thumb and the index finger was computed for every frame. For the hand grasp task, the Euclidean distance in pixels between the wrist and the average of the tips of all fingers, except the thumb. For the pronation-supination task, we computed the vector resulting from subtracting the key points of the pinky finger and the thumb. This vector was then transformed into the polar coordinates to obtain the degrees of the rotation for every frame, with respect to the normalization frames, as explained above.

The time signals were pre-processed with a Butterworth low pass frequency filter. To select the frequency of the filter we computed the Fourier transform of each graph to calculate their frequency components. The frequency selected to perform the low pass filter was the highest frequency of the peaks that have an amplitude at least higher than one-fourth of the amplitude of the highest frequency peak.

**Figure 2:**
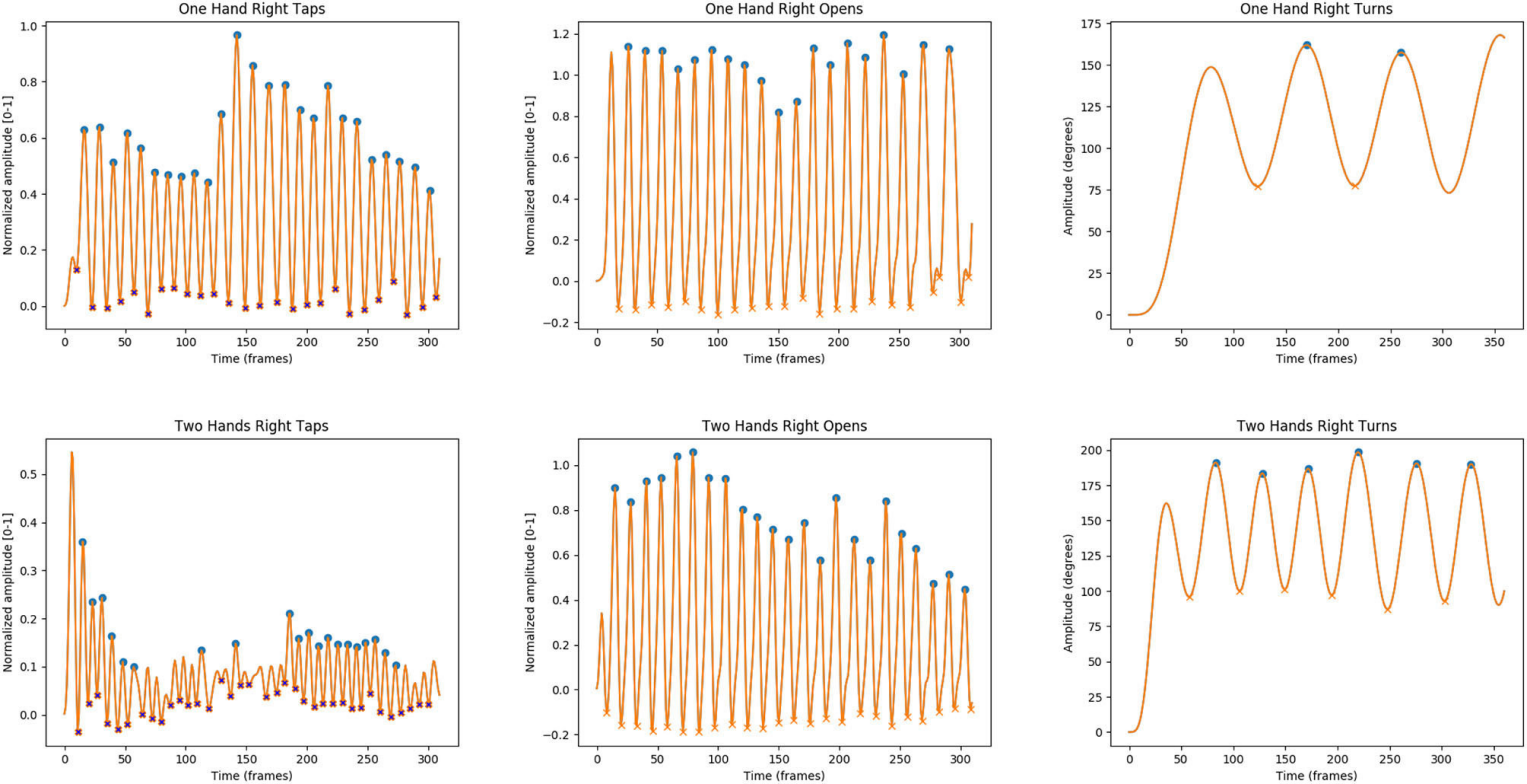
Acceleration traces during the single-hand (unilateral) and two-hand (bilateral) motor tasks using the webcam. Representative segment of the kinematic signal reconstructed during unilateral **(upper)** and bilateral **(bottom)** motor tasks from finger tapping, hand movement, and pronation/supination movements of the hand in a patient with Parkinson's disease (PD). Note the general worse performance in the dual tasks shown in the lower part of the image when compared with the corresponding task perform with just one hand.

In addition, we applied an amplitude correction, to eliminate the peaks due to noise. For the finger tapping task, we used a normalized pixel threshold of 0.1, while for the hand grasp and pronation/supination tasks, a 0.25 threshold was applied. After filtering every signal, we extracted the upper envelopes of the filtered signals, by detecting the peaks and interpolating among them (Figure 2). We extracted several features from the time curves: mean amplitude and SD of the peaks, speed (number of peaks per second), and fatigue (difference between the highest and the lowest values of the upper envelope of the curve). The three features were computed for both the left-hand and the right-hand tasks.

To confirm the accuracy of the time signals generated by our pipeline, the videos were assessed by an external clinician that manually labeled every landmark of the hand in videos of the finger tapping task of nine subjects. The clinician labeled the frames from each video of both hands and the software skipped two frames after each labeled frame. The salient points were automatically extracted for the remaining frames by the algorithm. Visual inspection of the salient points aligned to the original video were used to confirm the accuracy of the algorithm output.

### Classification Model Design

We designed a model to differentiate between the PD and HSs. For this, we extracted several single features that are known to be related with bradykinesia (mean amplitude, SD of the amplitude, speed, and fatigue) (19, 20) from both the hands either in the single-hand tasks and in the two-hands tasks. We trained three classifiers: Logistic Regression, Gaussian Naive-Bayes, and Random Forest. Each classifier received two values as input. These were the values of each feature for the left and right hands, respectively. We trained the classifiers using the features from each hand in the single hand tasks and using the features from each hand in the tasks performed with both hands simultaneously. A 4-fold cross-validation per classifier was applied and the receiver operating characteristic (ROC) curve per fold of the classifier was produced. Subsequently, an average ROC curve was calculated and the corresponding area under the curve (AUC) was used to compare the performance of the three candidate classifiers per classification task.

### Validation Dataset

An external validation dataset included 12 videos (six videos from the patients with PD and six from HSs) recorded with the same protocol. We evaluated the extracted features from each hand in the single-hand tasks, and the extracted features from each hand in the two-hand tasks.

### Statistical Analysis

We compared the demographic characteristics between the PD and HSs groups using Mann–Whitney's *U* non-parametric test (continuous) and the chi-square test (categorical). The extracted motor features from the videos of the finger tapping, hand movement, and pronation-supination movements of the hands stratified by side were compared between the PD and HSs groups using the non-parametric tests (Mann–Whitney test). Spearman's r correlations between the bradykinesia MDS-UPDRS-III sub-scores and the quantitative assessment methods were calculated. To rule out the confounding effects due to gender and hand size, we evaluated the performance of the classifiers on a strata containing only the male participants. The significance level was set at a 2-sided *P*-value of 0.05 and RStudio version 1.1.414 was used for the statistical analysis.

## Results

### Cohort Characterization

Twenty-two patients with PD (median [range] age: 49.7 [46.8–62] years) and 20 age-matched HSs (age: 49.9 [43.5–50.9] years) were enrolled in the study. The demographic features of the PD and HSs are described in Table 1. The patients with PD had a median disease duration of 2.6 [1.57–3.8] years since diagnosis and a median MDS-UPDRS-III score in Off-state of 18 [14–33] points.

**Table 1:** The demographic characteristics and clinical features of the patients with Parkinson's disease (PD).

		**Parkinson's disease** **(***n*** = 22)**	**Healthy subjects** **(***n*** = 20)**	***P*****-value** **(PD vs. HS)**
**Baseline demographic characteristics**				
Age, median (IQR)		49.7 (46.8–62)	49.9 (43.5–50.9)	0.21
Sex, *N* (%)	Man	16 (72.7)	6 (30)	0.01
	Woman	6 (27.3)	14 (70)	
Education, years median (IQR)		19 (17–20)	18 (16.7–20)	0.98
**Laterality Preference Index (LPI)**				
Handedness, *N* (%)	Right	19 (86.4)	20 (100)	0.48
	Left	2 (9.1)	0 (0)	
**Parkinson's disease patients' characteristics**				
Time since diagnosis (years), median (IQR)		2.6 (1.57–3.8)		
Predominant side at onset, *N* (%)	Right	17 (77.3)		
	Left	5 (22.7)		
Hoehn & Yahr stage, *N* (%)	Unilateral	19 (86.4)		
	Bilateral	3 (13.6)		
MDS-UPDRS III, median (IQR)		18 (14–33)		

### Quantitative Motor Assessment Using the Webcam

The single features extracted by the classifiers showed differences between the most affected side (MAS) and less affected side (LAS) in the patients with PD and between the dominant side (DS) and non-dominant side (NDS) according to handedness in HSs (Table 2). There were statistically significant differences for the unilateral finger tapping speed, hand movement speed, and pronation-supination movement amplitude (*P* < 0.05) (Table 2). For the bilateral task, there were statistically significant differences for the finger tapping amplitude, finger tapping speed, and hand movement speed (*P* < 0.05) (Table 2).

**Table 2:** Video-extracted motor features in the unilateral and bilateral tasks of the MDS-UPDRS-III bradykinesia upper limb motor tasks.

		**PD**		**HS**		
		**MAS**	**LAS**	**DS**	**NDS**	***P*** **-value**
**Finger tapping**						
Single hand (unilateral)	Amplitude Normalized amplitude [0–1]	0.73 (0.3)	0.81 (0.3)	0.84 (0.4)	0.87 (0.4)	0.345
	Speed (Time[frames])	2.03 (0.85)	2.26 (0.99)	2.35 (0.86)	2.19 (0.69)	0.247
	Fatigue	0.10 (0.27)	0.10 (0.27)	0.08 (0.15)	0.10 (0.19)	0.760
Two-hands (bilateral)	Amplitude Normalized amplitude [0–1]	0.56 (0.25)	0.77 (0.28)	0.79 (0.44)	0.80 (0.37)	0.042
	Speed (Time[frames])	2.13 (0.91)	2.25 (0.80)	2.03 (0.71)	2.03 (0.71)	0.705
	Fatigue	0.11 (0.20)	0.15 (0.25)	0.00 (0.33)	0.05 (0.33)	0.106
**Hand movements**						
Single hand (unilateral)	Amplitude Normalized amplitude [0–1]	0.92 (0.21)	0.97 (0.24)	0.9 (0.19)	1.02 (0.20)	0.904
	Speed (Time[frames])	1.40 (0.46)	1.80 (0.55)	1.68 (0.72)	1.58 (0.48)	0.143
	Fatigue	0.01 (0.18)	0.07 (0.18)	0.04 (0.16)	0.05 (0.19)	0.531
Two-hands (bilateral)	Amplitude	0.85 (0.25)	1.04 (0.27)	0.94 (0.15)	0.94 (0.15)	0.176
	Speed (Time[frames])	1.52 (0.41)	1.58 (0.46)	1.43 (0.41)	1.43 (0.41)	0.487
	Fatigue	−0.06 (0.17)	−0.01 (0.21)	0.11 (0.14)	0.09 (0.19)	0.044
**Pronation supination movement of the hand**						
Single hand (unilateral)	Amplitude (degrees)	116.67 (34.08)	137.21 (25.94)	136.18 (28.18)	120.72 (32.50)	0.053
	Speed (Time[frames])	1.55 (0.78)	1.78 (0.66)	1.65 (0.86)	1.54 (0.81)	0.698
	Fatigue	4.91 (24.01)	23.39 (36.64)	17.21 (53.79)	6.81 (39.19)	0.346
Two-hands (bilateral)	Amplitude (degrees)	113.07 (30.70)	130.50 (39.93)	128.45 (37.67)	129.85 (37.90	0.159
	Speed (Time[frames])	1.38 (0.46)	1.47 (0.47)	1.45 (0.69)	1.56 (0.70)	0.689
	Fatigue	21.94 (40.28)	4.88 (45.59)	28.39 (42.69)	2.00 (37.79)	0.622

*Video-extracted motor features in the unilateral and bilateral tasks of the MDS-UPDRS-III bradykinesia upper limb motor tasks. PD, Parkinson's disease; HSs, healthy subjects; MAS, most affected side; LAS, less affected side; DS, dominant side; and NDS, non-dominant side*.

### Concurrent Validity With MDS-UPDRS Part III Scores and Inertial Data

In the patients with PD, the video-extracted speed of the MAS showed significant moderate negative correlation with the MDS-UPDRS-III score of the MAS for hand movement (r = −0.50, *P* < 0.05). The video-extracted features showed correlation with objective quantification measures by IMU. Thus, the amplitude and the speed of movement of the MAS measured by the webcam showed significant moderate negative correlation with the corresponding MDRS-UPDRS-III bradykinesia item task measured by IMUs: finger tapping (r = −0.59, *P* < 0.05 and r = −0.51, *P* < 0.05 for amplitude and speed, respectively), hand movement (r = −0.50, *P* < 0.05; r = −0.70, *P* < 0.05), and pronation-supination movement of the hand (r = −0.67, *P* < 0.05; r = −0.66, *P* < 0.05).

### Classification Performance of the Developed Model: Differentiating PD From HSs Using the Webcam

In the developed model, the features extracted from single-hand tasks (herein named unilateral) overall showed the highest classification performance compared with those using features extracted from the two-hand tasks (herein named bilateral). The unilateral classifiers showed the mean sensitivity and specificity values ranging from 41 to 73% and 23 to 80%, respectively, for all the three features, with a cross-validation AUC ranging from 0.35 to 0.81 (Supplementary Table 1). In the unilateral motor tasks, the combined right-left speed feature for finger tapping and hand movement and the variation in the amplitude for the hand movements and pronation-supination movements of the hand showed the highest values and consistency across the three different classifiers (Supplementary Table 1). In the bilateral motor tasks, the combined right-left amplitude for finger tapping, hand movement, and the combined right-left amplitude variability for hand movements and fatigue for the hand movements and pronation-supination movement of the hands showed the highest value and consistency across the three classifiers (Supplementary Table 1). The stratified analysis among the male participants showed a cross validation AUC ranging from 0.21 to 0.88. In the unilateral motor tasks, the combined right-left speed feature for finger tapping and hand movement and the mean amplitude and variation in the amplitude for the pronation-supination movements of the hand showed the highest values and consistency in the three different classifiers. Similar results were found for the bilateral tasks. The performance of classifiers can be found in Supplementary Table 2.

### Validation of the Model on an Independent Dataset

**Figure 3:**
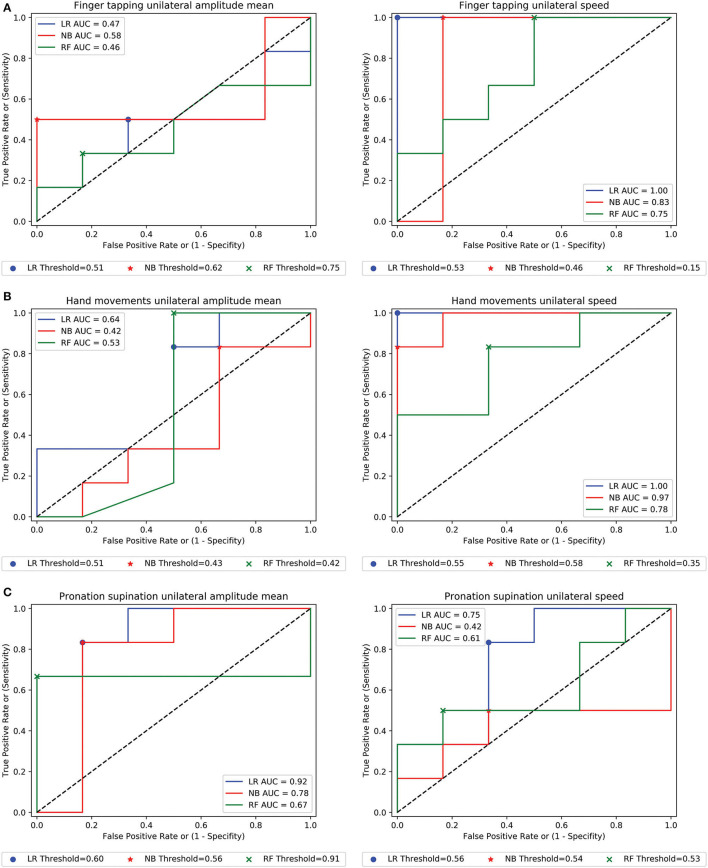
The receiver operating characteristic (ROC) curves in the validation cohort. **(A–C)** Example of ROC curves of the combined right-left amplitude and speed of movement in the single-hand (unilateral) motor tasks for the three used classifiers for the patients with PD classification.

When we applied our predictive model to an external validation dataset containing 12 videos (six PD and six HSs), in the unilateral motor tasks, the combined right-left speed for finger tapping, hand movement, and pronation-supination movement of the hand had the highest values and consistency across the three different classifiers, along with the combined right-left amplitude of the pronation-supination movement of the hand (Table 3 and Figure 3). The AUC range for the Logistic Regression model in the unilateral finger tapping, hand movement, and pronation-supinations tasks ranged from 0.47 to 1, 0.28 to 1.00, and 0.40 to 0.94, respectively. For Naïve Bayes model, the AUC results for the unilateral tasks ranged from 0.47 to 0.83 in the finger tapping, 0.22 to 0.97 for hand grasp, and 0.41 to 0.89 in the pronation supination task. Finally, for the Random Forest model, the AUCs ranged from 0.41 to 0.75 in the finger tapping, from 0.49 to 0.78 in the hand grasp, and from 0.61 to 0.89 in the pronation-supination task (Table 3). For the bilateral motor tasks, the combined right-left amplitude for finger tapping, pronation-supination movement of the hand, and the combined right-left amplitude variability for hand movements and speed for pronation-supination movement of the hands showed the highest value and consistency across the three classifiers (Table 3 and Figure 3).

**Table 3:** Model performance validation for PD classification.

**Validation dataset**		**LR**		**NB**		**RF**	
		**AUC**	**ACC**	**AUC**	**ACC**	**AUC**	**ACC**
**Finger tapping**							
Single hand	Amplitude_mean	0.472	0.333	0.583	0.500	0.458	0.500
(unilateral)l	Amplitude_std	0.583	0.500	0.472	0.500	0.458	0.417
	Speed	**1.000**	**1.000**	**0.833**	**0.667**	**0.750**	**0.667**
	Fatigue	**0.722**	**0.583**	**0.611**	**0.583**	0.417	0.500
Two-hands	Amplitude_mean	**0.777**	**0.750**	**0.750**	**0.500**	0.556	0.417
(bilateral)	Amplitude_std	0.444	0.500	0.500	0.583	0.597	0.583
	Speed	0.208	0.500	0.125	0.250	0.458	0.417
	Fatigue	0.500	0.416	0.222	0.500	0.556	0.500
**Hand movements**							
Single hand	Amplitude_mean	**0.639**	**0.667**	0.417	0.500	0.528	0.583
(unilateral)	Amplitude_std	0.278	0.500	0.222	0.333	0.486	0.417
	Speed	**1.000**	**1.000**	**0.972**	**0.917**	**0.778**	**0.583**
	Fatigue	0.472	0.417	**0.750**	**0.583**	0.556	0.583
Two-hands	Amplitude_mean	**0.750**	**0.667**	0.528	0.667	0.292	0.250
(bilateral)	Amplitude_std	0.472	0.500	0.528	0.417	**0.708**	**0.667**
	Speed	0.389	0.417	0.389	0.500	0.056	0.250
	Fatigue	0.333	0.417	0.500	0.417	0.569	0.583
**Pronation-supination movement of the hands**							
Single hand	Amplitude_mean	**0.917**	**0.667**	**0.778**	**0.750**	**0.667**	**0.417**
(unilateral)	Amplitude_std	**0.944**	**0.917**	**0.889**	**0.917**	**0.889**	**0.833**
	Speed	**0.750**	**0.750**	0.417	0.250	**0.611**	**0.667**
	Fatigue	0.444	0.417	**0.611**	**0.500**	**0.778**	**0.750**
Two-hands	Amplitude_mean	**0.833**	**0.750**	**0.861**	**0.833**	0.542	0.500
(bilateral)	Amplitude_std	**0.611**	**0.750**	**0.861**	**0.750**	**0.625**	**0.583**
	Speed	**0.611**	**0.583**	**0.750**	**0.750**	**0.750**	**0.750**
	Fatigue	0.306	0.333	0.583	0.583	0.306	0.500

*Validation results of the combined right and left motor features for PD classification. The validation was made using three classifiers: Logistic regression (LR), Gaussian Naïve-Bayes (NB), and Random Forest (RF). Features with cross-validation AUC > 0.6 are highlighted in bold. Units: normalized amplitude [0–1] for finger tapping and hand movements; amplitude (degrees) for pronation supination for amplitude features. Time (frames), for speed in all the tasks. AUC, cross-validation area under curve; ACC, accuracy*.

## Discussion

In this study, we have proven the concept that the motor performance can be assessed objectively using a conventional webcam. We found differences in the motor performances between the different sides of the body and between the patients with PD and HSs in all the upper limb motor tasks used to evaluate bradykinesia. The best performing classifiers were unilateral finger tapping and hand movement speed achieving an almost perfect classification similar to other diagnostic tests in PD (21). In addition to this high classification performance, the model correlated both with IMUs for quantitative assessment of motor function and the most used standard MDS-UPDRS part III, hence demonstrating concurrent validity with the existing gold standard methods.

### Video-Based Motor Evaluation

Different video-based systems have been described for the automated assessment of upper limb motor performance in the patients with PD (10). Most of these studies are restricted to the performance of one single motor task of the MDS-UPDRS-III with scarce comparisons of the motor performance between the different body sides (10, 11). Additionally, most of these systems have been traditionally used in a lab setting and have not yet been transitioned to the home setting (10, 22).

Recently, some video-based technologies have approached the study of motor performance using the low-quality video cameras. Using the smartphones cameras, different studies showed that it is possible to predict the presence of bradykinesia while performing the finger-tapping tasks, with an over 0.70 test accuracy, but with low discriminative capacity between the PD and HSs (23–25). Other studies, assessing the finger tapping motor task with conventional webcams also showed similar results to our study (11, 22).

One of the most interesting aspects of this work is that the performance of the model was lower when we evaluated the different motor features extracted individually than when we integrated them. Specially, the inclusion of the side reflecting the asymmetry typical of PD increased the diagnostic performance dramatically and emphasized how critical the integration of information is for the clinical diagnosis.

The integration of information happens during a standard clinical evaluation. To evaluate a patient and establish a diagnosis, the neurologist needs to: (1) have a predefined criteria of average and non-average motor performance for each body side, (2) evaluate the different aspects of the motor performance (e.g., speed, amplitude, and fatigue), (3) compare the motor performance between each body side, (4) evaluate if the observed pattern matches the previous cases with the disease or that of the HSs. In our study, we replicated this behavior and trained a model that can recognize decrements in amplitude and speed of movement between the PD and HSs groups. This information was also combined with the motor performance asymmetry of one side compared with the other one. Thus, the accuracy obtained for some of the extracted motor features (i.e., finger tapping speed or hand grasp speed) could complement the one obtained from an expert neurologist (26). It is remarkable that the computer needed a 12 s video per task to perform a classification of PD vs. HSs based on the performance of bradykinesia tasks. Therefore, our method has many conceptual resemblances with the routine diagnostic process, making it interpretable and aligned with the standard of care, and supporting the potential of feature integration mimicking the behavior of the human brain.

Importantly, the system could also detect the differences in motor performance between the unilateral tasks and bilateral tasks, showing a worse performance when performing bilateral tasks in the group of patients with PD compare with HSs (Table 2). This is in line with the described impaired ability to perform the bilateral tasks, either simultaneously or sequentially, that occurs in the patients with PD. Thus, when performing a bilateral task, the motor performance in the patients with PD can show a dramatic reduction in the movement of the most affected side compared to the unilateral tasks (27, 28).

This proof-of-concept study hence demonstrates that a standard webcam coupled with an artificial intelligence method has potential for accurately assessing upper limb bradykinesia in the patients with PD remotely. The camera employed for all the experiments was a webcam of standard laptop produced in 2010 (a 2010 MacBookPro). Any current laptop or mobile device allows video recording with a higher resolution and frame rate. This tool expands the portfolio of technologies available for evaluating the patients with PD, with the advantages of not needing any dedicated equipment outside of a standard laptop and using a video which also permits a simultaneous verification of the motor performance using the traditional clinical methods (i.e., the healthcare professional could review the raw video that generated the score when needed for quality control purposes). The most salient applications of this video-based technology could be the use in remote teleconsultations, which have surged with the severe acute respiratory syndrome coronavirus 2 (SARS-CoV-2) pandemic (29) and the decentralized clinical trials, as a complement for the standard assessments using MDS-UPDRS or other objective evaluation methods (i.e., sensors) (30, 31). In addition, this system could help minimize variability in clinical assessment in the clinical trials.

In this way, and in keeping with the previous initiatives (11, 22), we have recently developed a web app for the remote recording of the upper limb bradykinesia motor tasks. The app includes conformance statement signing, video instructions for every task, and the indications to facilitate hand positioning. Furthermore, it requires no software to be installed, thus using any standard laptop and any operating system. The upcoming studies will show the feasibility of its implementation for the assessment of the tasks in the at-home setting.

### Limitations

Our work has several limitations. First, this is a proof-of-concept study with a reduced sample size, with specific disease characteristics (age and duration of disease), and the comparison was made only with healthy controls. Both the factors limit the generalizability of the results. Additionally, the study cohort is integrated by early patients with PD with predominant unilateral motor symptoms. Yet, this provides a convenient scenario to test the discriminative strength of the method. In the future, our results should be verified in larger cohorts with a representation of a broad spectrum of the patients with PD, such as the groups with different age and disease duration. In addition, the discriminative power of the method when including other parkinsonian syndromes in the mix remains to be established as well. Another limitation is that we restricted the assessment to the upper limb bradykinesia motor tasks of the MDS-UPDRS III. However, the motor performance of upper limbs is a predictive characteristic of onset and PD progression (32, 33). Therefore, its analysis is of the utmost relevance for the clinical evaluation and outcome of the patients with PD. The assessment of the global motor performance, such as lower limbs, and other disease manifestations, such as tremor, axial signs, and gait represents a future expansion of the current concept of objectively measuring other disease motor features using a standard video. This can improve the model performance and hence needs to be investigated. Finally, we focused our analysis on the evaluation of the binary classification performance of the present method. Future work should evaluate the additional quantitative aspects of the motor performance, increasing the granularity of the information, be able to rate the disease severity, and the detection of subtle changes in the motor status along the time, and after a therapeutic intervention. Those aspects will be critical for the application of this system in telemedicine and potentially clinical trials.

## Conclusions

We proved the concept that a novel webcam-based technology can accurately evaluate bradykinesia, the single core feature that allows the diagnosis of parkinsonism, in a remote setting, using artificial intelligence. This method has an accuracy performance that could complement the usual diagnostic process performed by the experienced movement disorders specialists and could be easily expanded to other disease manifestations. Our results need to be confirmed in the larger studies, such as patients with other forms of parkinsonism, age groups, and disease status.

## Data Availability Statement

The raw data supporting the conclusions of this article will be made available by the authors, without undue reservation.

## Ethics Statement

The studies involving human participants were reviewed and approved by the Ethics Committee of HM Hospitales who approved the study protocol (Protocol Number: 18.05.1245-GHM). The patients/participants provided their written informed consent to participate in this study.

## Author Contributions

MHGM has contributed to the design of the solution, the recruitment and evaluation of the participants, the analysis of the data, and the manuscript draft. SD, JV-O, and NM have participated in the technology development, the analysis, and the draft of the manuscript. AA and TM have participated in the critical review of the project, evaluation of the results, and the manuscript elaboration. AS-F has contributed to the conceptualization of the solution, its development, the clinical validation, the draft, and the review of the manuscript. All authors contributed to the article and approved the submitted version.

## Funding

This study was supported by the Joint Program for Neurodegenerative Diseases and the Instituto de Salud Carlos III (Reference Number: HESOCARE-329-073) as part of the iCARE-PD consortium.

## Conflict of Interest

MHGM has received speaker and travel honoraria from Novartis Pharmaceutical. AS-F has received funding from Consejería de Educación, Juventud y Deporte of Comunidad de Madrid and the People Programme (Marie Curie Actions) of the European Union's Seventh Framework Programme (FP7/2007-2013) under Research Executive Agency Grant 291820. He was also funded by ERA-NET Horizon 2020 program JPCOFUND2 (Reference Number: HESOCARE-329-073) and has also received speaker and travel honoraria from Teva, Zambon, Abbvie, and Novartis Pharmaceutical. He owns common stock in Leuko Labs, Inc., a company with commercial interests in a Medical Device developed for neutropenia detection. He was also an inventor of a Method and Apparatus for Motor Function characterization (US 2020/0060622 Al) that has been licensed to an independent commercial entity (nQ-Medical) by the Massachusetts Institute of Technology, Massachusetts, United States. TM received consultancies from nQ Medical and research funding from EU Joint Programme - Neurodegenerative Disease Research. The remaining authors declare that the research was conducted in the absence of any commercial or financial relationships that could be construed as a potential conflict of interest.

## Publisher's Note

All claims expressed in this article are solely those of the authors and do not necessarily represent those of their affiliated organizations, or those of the publisher, the editors and the reviewers. Any product that may be evaluated in this article, or claim that may be made by its manufacturer, is not guaranteed or endorsed by the publisher.

## Supplementary Material

Supplementary Figure 1 Landmarks extracted for every hand by OpePose.

Supplementary Table 1 Model performance training for Parkinson's disease (PD) classification. Classification results of the combined right and left motor features for PD classification prediction. The training of the model was made using three classifiers: Logistic regression (LR), Gaussian Naïve-Bayes (NB), and Random Forest (RF). Features with cross-validation AUC > 0.6 are highlighted in bold. Units: normalized amplitude [0–1] for finger tapping and hand movements; amplitude (degrees) for pronation supination for amplitude features. Time (frames), for speed in all tasks. AUC, cross-validation area under curve; ACC, accuracy.

Supplementary Table 2 Model performance training for PD classification in men. Classification results of the combined right and left motor features for PD classification prediction. The training was made using three classifiers: LR, NB, and RF. The features with cross-validation AUC > 0.6 are highlighted in bold. Units: normalized amplitude [0–1] for finger tapping and hand movements; amplitude (degrees) for pronation supination for the amplitude features. Time (frames), for speed in all tasks. AUC, cross-validation area under curve; ACC, accuracy.
