# Supplementary figures and images for: Remote Evaluation of Parkinson's Disease Using a Conventional Webcam and Artificial Intelligence
Source: Front Neurol. 2021 Dec 23;12:742654. doi: 10.3389/fneur.2021.742654 (PMC8733479; doi:10.3389/fneur.2021.742654)

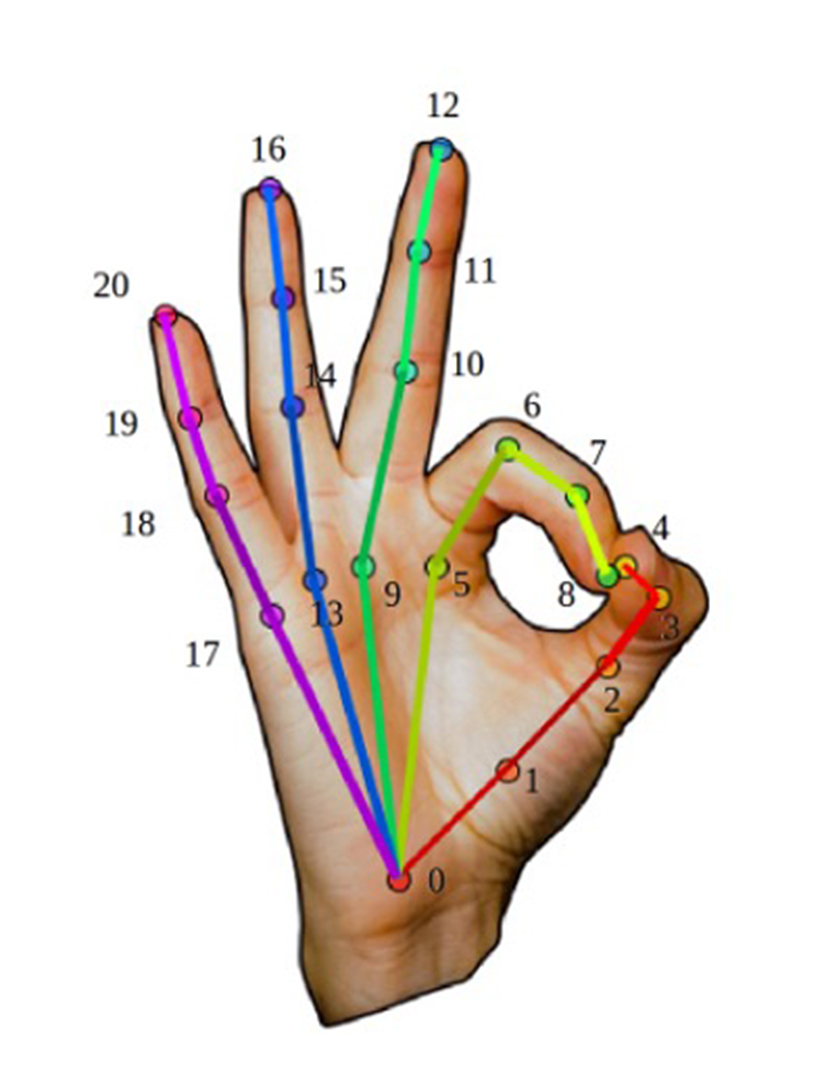

Supplement: Supplementary Figure 1 — Landmarks extracted for every hand by OpePose. [file Image_1.JPEG]
