# Supplementary material for: Remote Evaluation of Parkinson's Disease Using a Conventional Webcam and Artificial Intelligence
Source: Front Neurol. 2021 Dec 23;12:742654. doi: 10.3389/fneur.2021.742654 (PMC8733479; doi:10.3389/fneur.2021.742654)
Supplement: Supplementary Table 1 — Model performance training for Parkinson's disease (PD) classification. Classification results of the combined right and left motor features for PD classification prediction. The training of the model was made using three classifiers: Logistic regression (LR), Gaussian Naïve-Bayes (NB), and Random Forest (RF). Features with cross-validation AUC > 0.6 are highlighted in bold. Units: normalized amplitude [0–1] for finger tapping and hand movements; amplitude (degrees) for pronation supination for amplitude features. Time (frames), for speed in all tasks. AUC, cross-validation area under curve; ACC, accuracy. [file Table_1.pdf]

Supplementary Table 1. Model performance training for Parkinson's disease classification

Model performance		LR		NB		RF	
		**AUC**	**ACC**	**AUC**	**ACC**	**AUC**	**ACC**
**Finger tapping**							
Single hand (unilateral)l	Amplitude_mean	0.545	0.538	0.399	0.461	0.476	0.482
	Amplitude_std	0.351	0.405	0.355	0.438	0.471	0.481
	Speed	0.550	0.535	0.569	0.518	**0.673**	**0.614**
	Fatigue	0.369	0.415	0.521	0.468	0.503	0.497
Two-hands (bilateral)	Amplitude_mean	**0.634**	**0.567**	0.506	0.531	0.402	0.44
	Amplitude_std	0.346	0.401	0.434	0.479	0.359	0.401
	Speed	0.427	0.465	0.433	0.409	0.528	0.509
	Fatigue	0.597	0.588	0.532	0.536	0.522	0.509
**Hand movements**							
Single hand (unilateral)l	Amplitude_mean	0.427	0.435	0.320	0.365	0.291	0.324
	Amplitude_std	**0.663**	**0.675**	**0.651**	**0.620**	**0.615**	**0.567**
	Speed	**0.705**	**0.646**	0.526	0.520	0.558	0.567
	Fatigue	0.352	0.379	0.547	0.517	0.462	0.454
Two-hands (bilateral)	Amplitude_mean	0.576	0.582	**0.616**	**0.575**	0.389	0.43
	Amplitude_std	**0.679**	**0.675**	**0.640**	**0.617**	0.593	0.595
	Speed	0.482	0.529	0.467	0.455	0.518	0.513
	Fatigue	**0.715**	**0.637**	**0.729**	**0.629**	**0.738**	**0.651**
**Pronation-supination movement of the hands**							
Single hand (unilateral)l	Amplitude_mean	**0.692**	**0.636**	0.587	0.525	0.553	0.535
	Amplitude_std	**0.793**	**0.709**	**0.807**	**0.745**	**0.807**	**0.715**
	Speed	0.486	0.507	0.467	0.469	0.481	0.507
	Fatigue	0.497	0.514	**0.620**	**0.561**	0.449	0.444
Two-hands (bilateral)	Amplitude_mean	0.486	0.494	0.437	0.464	**0.605**	**0.603**
	Amplitude_std	0.590	0.545	0.525	0.556	0.489	0.495
	Speed	0.340	0.385	0.533	0.560	0.409	0.444
	Fatigue	0.594	0.573	**0.617**	**0.565**	**0.786**	**0.732**

Classification results of the combined right and left motor features for Parkinson's disease classification prediction. The training of the model was made using three classifiers: Logistic regression (LR), Gaussian Naïve-Bayes (NB) and Random Forest (RF). Features with cross-validation AUC > 0.6 are highlighted in bold. Units: Normalized amplitude[0-1] for finger tapping and hand movements; Amplitude (degrees) for pronation supination for amplitude features.Time (frames), for speed in all tasks. AUC, cross-validation area under curve; ACC; accuracy
