# Supplementary material for: Remote Evaluation of Parkinson's Disease Using a Conventional Webcam and Artificial Intelligence
Source: Front Neurol. 2021 Dec 23;12:742654. doi: 10.3389/fneur.2021.742654 (PMC8733479; doi:10.3389/fneur.2021.742654)
Supplement: Supplementary Table 2 — Model performance training for PD classification in men. Classification results of the combined right and left motor features for PD classification prediction. The training was made using three classifiers: LR, NB, and RF. The features with cross-validation AUC > 0.6 are highlighted in bold. Units: normalized amplitude [0–1] for finger tapping and hand movements; amplitude (degrees) for pronation supination for the amplitude features. Time (frames), for speed in all tasks. AUC, cross-validation area under curve; ACC, accuracy. [file Table_2.pdf]

Supplementary Table 2. Model performance training for Parkinson's disease classification in males

Model in males		LR		NB		RF	
		AUC	ACC	AUC	ACC	AUC	ACC
**Finger tapping**							
Single hand (unilateral)l	Amplitude_mean	0.508	0.692	0.570	0.662	0.551	0.682
	Amplitude_std	**0.681**	**0.688**	0.562	0.734	0.433	0.536
	Speed	**0.663**	**0.652**	**0.732**	**0.736**	**0.723**	**0.724**
	Fatigue	0.482	0.690	0.514	0.552	0.485	0.580
Two-hands (bilateral)	Amplitude_mean	0.492	0.682	0.532	0.718	0.362	0.574
	Amplitude_std	**0.808**	**0.686**	**0.831**	**0.884**	**0.768**	**0.802**
	Speed	0.468	0.700	0.597	0.560	0.464	0.650
	Fatigue	**0.714**	**0.726**	**0.870**	**0.848**	**0.672**	**0.710**
**Hand movements**							
Single hand (unilateral)l	Amplitude_mean	0.523	0.700	0.267	0.580	0.421	0.564
	Amplitude_std	0.268	0.698	0.277	0.608	0.422	0.536
	Speed	**0.754**	**0.674**	0.473	0.726	0.283	0.556
	Fatigue	0.185	0.718	0.248	0.544	0.404	0.594
Two-hands (bilateral)	Amplitude_mean	0.448	0.696	**0.630**	**0.568**	0.553	0.584
	Amplitude_std	0.457	0.696	0.382	0.590	0.212	0.580
	Speed	0.406	0.706	0.543	0.576	**0.641**	**0.708**
	Fatigue	**0.779**	**0.706**	**0.623**	**0.640**	0.580	0.676
**Pronation-supination movement of the hand**							
Single hand (unilateral)l	Amplitude_mean	**0.888**	**0.800**	**0.905**	**0.726**	**0.709**	**0.752**
	Amplitude_std	**0.788**	**0.696**	**0.917**	**0.786**	**0.816**	**0.848**
	Speed	0.363	0.694	0.464	0.548	0.506	0.584
	Fatigue	0.348	0.584	**0.726**	**0.674**	0.440	0.590
Two-hands (bilateral)	Amplitude_mean	**0.711**	**0.676**	**0.798**	**0.670**	**0.626**	**0.596**
	Amplitude_std	0.552	0.632	**0.810**	**0.682**	0.583	0.620
	Speed	0.405	0.690	**0.726**	**0.596**	0.481	0.614
	Fatigue	0.412	0.614	**0.631**	**0.694**	**0.726**	**0.698**

Classification results of the combined right and left motor features for Parkinson's disease classification prediction. The training was made using three classifiers: Logistic regression (LR), Gaussian Naïve-Bayes (NB) and Random Forest (RF). Features with cross-validation AUC > 0.6 are highlighted in bold. Units: Normalized amplitude[0-1] for finger tapping and hand movements; Amplitude (degrees) for pronation supination for amplitude features.Time (frames), for speed in all tasks. AUC, cross-validation area under curve;ACC; accuracy
